# Inflammation and Barrier Function Deficits in the Bladder Urothelium of Patients with Chronic Spinal Cord Injury and Recurrent Urinary Tract Infections

**DOI:** 10.3390/biomedicines10020220

**Published:** 2022-01-20

**Authors:** Shu-Yu Wu, Yuan-Hong Jiang, Jia-Fong Jhang, Yung-Hsiang Hsu, Han-Chen Ho, Hann-Chorng Kuo

**Affiliations:** 1 Department of Urology, Taipei Tzu Chi Hospital, Buddhist Tzu Chi Medical Foundation, New Taipei City 23142, Taiwan; nobookrain2014@gmail.com; 2 Department of Urology, School of Medicine, Tzu Chi University, Hualien 97004, Taiwan; redeemerhd@gmail.com (Y.-H.J.); alur1984@tzuchi.com.tw (J.-F.J.); 3 Department of Urology, Hualien Tzu Chi Hospital, Buddhist Tzu Chi Medical Foundation, Hualien 97004, Taiwan; 4 Department of Pathology, Hualien Tzu Chi Hospital, Buddhist Tzu Chi Medical Foundation, Hualien 97004, Taiwan; yhhsu@mail.tcu.edu.tw; 5 Department of Pathology, School of Medicine, Tzu Chi University, Hualien 97004, Taiwan; 6 Department of Anatomy, School of Medicine, Tzu Chi University, Hualien 97004, Taiwan; hcho@gms.tcu.edu.tw

**Keywords:** spinal cord injury, urothelial dysfunction, regenerative deficits, recurrent urinary tract infection

## Abstract

Patients with spinal cord injury (SCI) commonly experience neurogenic voiding dysfunctions and urinary tract complications, including recurrent urinary tract infections (rUTI). The bladder mucosa barrier function contributes to UTI prevention. This study investigated changes in bladder urothelium protein expression in patients with SCI and rUTI. From June 2011 to November 2017, 23 patients (19 men and 4 women) with chronic SCI were enrolled (mean age: 43 years. Bladder tissues from 6 healthy adults served as the normal control group. Biopsy samples (9 partial cystectomies and 14 bladder biopsies) were analyzed for functional biomarkers using western blot and immunohistochemistry analysis. The barrier function proteins E-cadherin, zonula occludens 1 (ZO-1) and uroplakin III (UPK-3) were significantly reduced, whereas tumor protein p63 (TP63) was significantly increased in SCI patients compared with controls. No significant differences in basal cell progenitor proteins were observed between groups. The proliferation marker Ki-67, the proapoptotic marker BCL-2-associated X protein (BAX), and proinflammatory proteins were increased in patients with SCI compared with controls. No significant differences were observed between SCI patients with and without recently rUTI. These results suggest that SCI patients experience chronic bladder inflammation, increased apoptosis, and reduced barrier function, contributing to rUTI.

## 1. Introduction

Spinal cord injury (SCI) is a global disease. The estimated prevalence is 0.07–0.1% in different countries [1,2]. The most common reason for SCI is trauma, and more than 70% SCI patients suffer from multiple injuries [3]. Nearly 80% of SCI patients report some degree of bladder dysfunction within 1 year after injury [4,5]. The SCI can interrupt the signaling between pontine micturition center and the lower urinary tract, resulting in neurogenic lower urinary tract dysfunction. Urinary dysfunction has a significant clinical, physical, and quality of life burden in patients with SCI and neurogenic bladder [6]. These patients might present with detrusor underactivity, detrusor overactivity, or detrusor sphincter dyssynergia (DSD) in urodynamic studies. Recurrent urinary tract infections (rUTI) are among the most common complications and are often difficult to treat. rUTI can lead to increasingly serious urinary incontinence, frequent catheter obstruction, poor quality of life and even mortality [7,8]. In recent studies, 22% of patients with acute SCI developed a UTI during the first 50 days following SCI, and the incidence of UTI among patients with chronic SCI was 0.68 per 100 patients daily. The risk factors known to lead to UTI include the performance of invasive procedures without antibiotic prophylaxis, cervical SCI, and chronic catheterization. Complete functional dependence and vesicoureteral reflux are factors known to be associated with rUTI [9,10].

Traditionally, UTI was associated with female sex, sexual intercourse, urinary incontinence, increased postvoid urine volume, urine catheterization, poor voiding function, abnormal urinary tract anatomy, and poor host immunity [11]. Recently, the barrier function of the bladder urothelium was considered as important factor associated with the occurrence of rUTI. SCI causes changes in the urothelial morphology and barrier function, leading to the development of chronic inflammation and increased susceptibility to UTI due to a yet unspecified neural-mediated pathway [12,13]. The relationship between SCI and their bladder function plays an important role in the occurrence of rUTI.

In addition to barrier function, the bladder urothelium was shown to feature both sensory and transducer properties [8]. In recent animal studies, bladder barrier function was successfully re-established after tight junction disruption, which resulted in changes in the urothelium morphology. The increased expression of sensory receptors and the increased release of excitatory mediators were observed following SCI [14,15,16]. Based on current evidence, SCI and rUTI may share same molecular reaction in some aspects but there are also some differences. However, no published studies examined differences in bladder functions between patients with SCI with and without rUTI and healthy controls. Our study explored changes in urothelial dysfunction in patient with chronic SCI and rUTI, which could provide a better understanding of rUTI in SCI patients and enhance patient care.

## 2. Materials and Methods

From June 2011 to November 2017, 23 patients with chronic SCI and neurogenic bladder were enrolled in this study. These patients were preparing for onabotulinumtoxinA detrusor muscle injections or augmentation enterocystoplasty procedures due to urinary incontinence caused by severe detrusor overactivity. Traditional conservative therapies and antimuscarinic medications were previously applied with poor results. All of these patients had upper motor neuron lesions of the bladder, with either cervical- or thoracic-level injuries. All patients were able to void by reflex voiding plus temporary clean intermittent catherization (CIC) without requiring long-term indwelling catheter. The recurrent UTI was defined as at least three times of UTI episode per year or twice in 6 months. According to the patients’ past histories, all included patients with SCI were previously treated for symptomatic rUTI, and some but not all patients had recent rUTI that occurred within one month before the surgical procedure. A symptomatic patient with a positive urine culture and cured by correct antibiotics therapy was defined as a real UTI episode. Patients were then grouped into those with recent rUTI and those without recent rUTI according to their history, urinalysis results, and urine culture outcomes before the operation. Video-urodynamic studies (VUDS) were performed preoperatively in every patient to verify detrusor function, with or without DSD, and to identify bladder outlet obstructions or intrinsic sphincter deficiencies.

Bladder biopsy samples were collected from all participants (9 partial cystectomies and 14 bladder biopsies), and protein levels were analyzed using standard western blot procedures and immunohistochemistry staining analyses. Additionally, 6 healthy adults (without SCI) served as the normal control group. The specimens were obtained from transurethal prostate resection, urethroscopic exam, and cystoscopy before a sling surgery. Their medical records were checked completely and confirmed that there is no UTI. Preoperative VUDS and urinalysis were performed, proving normal bladder functionality and no UTI. Cold cup bladder biopsies were obtained during the onabotulinumtoxinA detrusor muscle injections in patients with SCI and during transurethral procedures in the control group. Specimens were obtained from the posterior bladder wall, approximately 2 cm above the bladder trigone. In patients who received augmentation enterocystoplasty procedures, a partial cystectomy of the bladder dome was performed before bladder augmentation to obtain bladder specimens. Tissue samples were assessed by the pathology department to exclude the possibility of malignancies, and additional tissue specimens were embedded in optimal cutting temperature medium and stored at −80 °C for further investigation.

The levels of bladder urothelial proteins were examined, including the barrier and junction proteins E-cadherin, zonula occludens-1 (ZO-1), and uroplakin III (UPK-3); the urothelial differentiation proteins cytokeratin (CK)-20, CK-14, CK-5 and tumor protein p63 (TP63); the basal cell progenitor proteins CD-34 and Sonic hedgehog (Shh); proliferation protein Ki-67; the proapoptotic proteins BCL-2-associated X protein (BAX), B-cell lymphoma 2 family protein (BCL-2), and P53; and the proinflammatory proteins tryptase, tumor necrosis factor-alpha (TNF-α) and transforming growth factor-beta (TGF-β).

The bladder biopsy specimens from patients with SCI and control individuals were homogenized in liquid nitrogen and lysed for 10 min on ice using HyTra Tissue Protein Extraction Reagent (Roche Diagnostics, Mannheim, Germany). The extraction solution was supplemented with a protease inhibitor cocktail (Roche Diagnostics) and a phosphatase inhibitor cocktail (Roche Diagnostics). Proteins were separated by electrophoresis on 12% Tris-glycine gel. After gel electrophoresis, the proteins were transferred to 0.2 µm polyvinylidene difluoride (PVDF) membranes, which were blocked in 3% skim milk for 1 h, followed by the addition of E-cadherin, ZO-1, UPK3, CK-20, CK-14, CK-5, TP63, CD34, Shh, Ki-67, BAX, BCL-2, P53, tryptase, TNF-α, and TGF-β primary antibodies and with glyceraldehyde 3-phosphate dehydrogenase (GAPDH) as the loading control. Details regarding antibody dilutions, antibody manufactures, and expected protein sizes are shown in Table 1. The membranes were incubated for overnight at 4 °C; washed 4 times for 10 min each time in Tris-buffered saline containing Tween 20 (TBST). The secondary antibody (horseradish peroxidase [HRP]-conjugated goat anti-rabbit IgG, diluted 1:5000, from Santa Cruz Biotechnology, Santa Cruz, CA, USA) was then applied. The membranes were finally probed with an enhanced chemiluminescence reagent (ECL; Millipore Corporation, Saint Paul, MN, USA) and exposed to X-ray films. Films were quantified using a gel documentation system (Quantity One Version 4.6.2, Bio-Rad Laboratories, Hemel Hempstead, Hertfordshire, UK). GAPDH was used as the loading control to normalize quantification. All bladder samples from all patients were analyzed with identical techniques, following procedures reported in our previous studies [17,18].

The immunohistochemistry staining procedures were performed as follows. Tissues sections were deparaffinized and rehydrated through gradient incubations in non-xylene, ethanol, distilled water, 3% H_2_O_2_ solution and TBST. Antigen retrieval was performed using citrate buffer (pH 6.0 or 8.0). The specimens were incubated with primary antibody, and signal detection was performed (UltraVision Quanto Detection System, from Thermo Fisher Scientific, MA, USA, catalog number: TL-060-QHD). Briefly, primary antibodies were applied to the tissue sections and incubated according to the manufacturer’s recommendations for 10 min. HRP Polymer Quanto was applied and incubated for 10 min after the buffer wash step. A 30 µL volume DAB Quanto Chromogen was added to 1 mL of DAB Quanto Substrate, mixed by swirling, and applied to tissue for a 5-min incubation. Tissue sections were washed with deionized water, counterstained, and cover slipped using a permanent mounting media. Immunofluorescent images were assessed using fluorescence microscopy and processed using a digital imaging system. The distribution and fluorescence intensity were obtained based on images obtained using a confocal microscope.

Data are expressed as the mean ± standard deviation. Differences in clinical parameters between patients with SCI with recent rUTI, patients with SCI without recent rUTI, and control individuals were analyzed using the independent T-test. A *p*-value < 0.05 was considered significant. All calculations were performed using SPSS for Windows (version 16.0, IBM, Chicago, IL, USA).

This study was approved by the Research Ethics Committee of Hualien Tzu Chi Hospital (IRB: 110-033-B, approval date 22 February 2021). Given the retrospective nature of this study, the requirement for informed consent was waived by the Research Ethics Committee of Hualien Tzu Chi Hospital. All methods used in this study were conducted in accordance with relevant guidelines and regulations.

## 3. Results

Among the 23 patients with chronic SCI, 19 were men and 4 were women; fifteen had high-level cervical SCI, and 8 had thoracic or lumbar SCI. The mean patient age was 43 ± 14.2 years, and the interval from the SCI episode to the time of bladder biopsy was 12.8 ± 11.9 years. All patients presented with urodynamic detrusor overactivity, and 15 (65.2%) patients had DSD. Eight patients had a recent rUTI, and 15 patients had a history of rUTI but no recent rUTI. The patients’ baseline urodynamic parameters are presented in Table 2.

**Figure 1:**
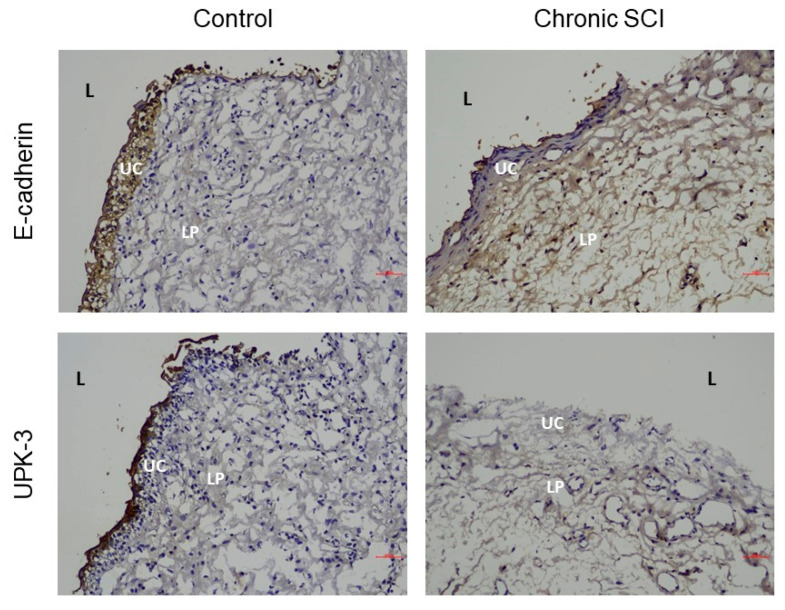
Immunohistochemistry showing barrier and junction proteins E-cadherin and uroplakin-III (UPK-3) in bladder tissues from chronic spinal cord injury (SCI) patients and control subjects. E-cadherin and UPK-3 are positive (brown) in superficial umbrella cells in control group but lack in SCI bladder. Labeling of lamina propria is unspecific. L, lumen; UC, umbrella cell; LP, lamina propria. Scale bars: 20 μm.

**Figure 2:**
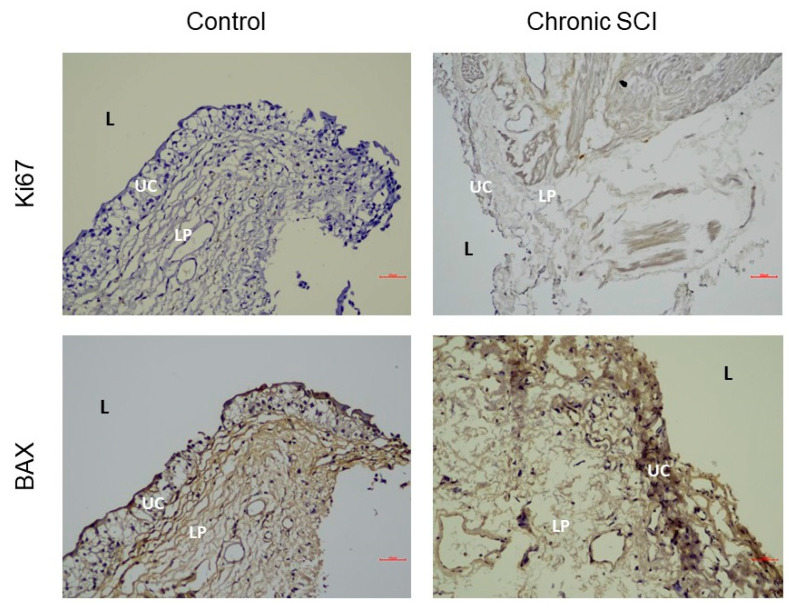
Immunohistochemistry showing proliferation protein Ki-67 and pro-apoptosis proteins (BAX) in bladder tissues of chronic spinal cord injury (SCI) patients and control subjects. Index of Ki67 is lower, making it difficult to identified differences with human eyes. BAX is positive in superficial umbrella cells in SCI group. Labeling of lamina propria is unspecific. L, lumen; UC, umbrella cell; LP, lamina propria. Scale bars: 20 μm.

**Figure 3:**
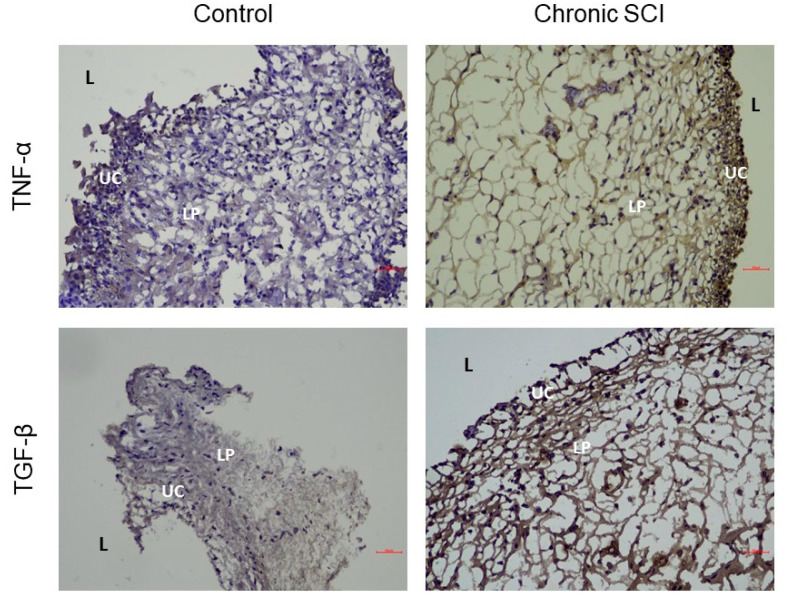
Immunohistochemistry showing inflammation proteins tumor necrosis factor-α (TNF-α) and transforming growth factor-β (TGF-β) in bladder tissues of chronic SCI patients and control subjects. TNF-α and TGF-β are more obvious in SCI group than control group. Labeling of lamina propria is unspecific. L, lumen; UC, umbrella cell; LP, lamina propria. Scale bars: 20 μm.

In the evaluation of barrier function, samples from patients with SCI showed significantly reduced expression levels of E-cadherin, ZO-1, and UPK-3 (Figure 1). Among the examined urothelial differentiation proteins, TP63 was the only significantly different protein, presenting increased levels in patients with SCI compared with controls. The basal cell progenitor proteins CK-20, CK-14, and CK-5 showed no significant differences between bladder samples from patients with SCI and those from controls (Table 3). The patients with SCI presented with increased levels of Ki-67 protein compared with that of controls (Figure 2). BAX was the only proapoptotic protein that was significantly increased in patients with SCI compared with controls (Figure 2). All proinflammatory proteins, including tryptase, TNF-α, and TGF-β were significantly increased in patients with SCI compared to the controls (Figure 3). However, no significant differences in any protein expression levels were observed between patients with SCI with and without recent rUTI. Details regarding the observed protein levels among groups are presented in Table 3. The protein expression analysis showed that bladder samples from patients with SCI had higher levels of apoptosis and inflammation markers, poor barrier function, and increased cell proliferation, regardless of whether the patients had experienced recent rUTI. However, no differences in basal cell proliferation or urothelial cell differentiation markers were observed in patients with SCI related to control patients.

## 4. Discussion

This study demonstrated a decrease in the expression levels of urothelial adhesion and junction proteins (E-cadherin, ZO-1, and UPK-3); increased expression of inflammatory proteins (tryptase, TNF-α, and TGF-β); and the increased expression of limited apoptosis (BAX), urothelial differentiation (TP63), and cell proliferation (Ki-67) markers in patients with chronic SCI, regardless of whether they had experienced a recent rUTI compared with controls. These results confirm the presence of increased suburothelial inflammation, defective urothelial barrier function, and a lack of basal cell regeneration in patients with chronic SCI, which might result in the development of rUTI in this population. This study provides new directions for subsequent treatment of such SCI patients in addition to antibiotics and improving their urination.

Recurrent bacterial cystitis is one of the most common lower urinary tract dysfunctions that occur in patients with SCI [19,20]. In a previous study of urothelial dysfunction among women with rUTI, we demonstrated that chronic inflammation, increased urothelial cell apoptosis, and barrier functional impairments in the urothelium might represent the underlying pathophysiology that results in rUTI [18]. Normal urothelial regeneration facilitates basal cell regeneration in the urothelium and differentiation following bacterial invasion, which promotes the exfoliation of apical urothelial cells to expel the bacteria infecting these cells [21]. Previous animal studies showed that following acute bladder injury, the urothelium is able to repair and form an intact bladder barrier within 72 h [22]. In patients with impaired urothelial regenerative function, the defense mechanism against bacterial invasion is typically insufficient, which allows pathogens to penetrate through the defective urothelium, inducing subsequent infection and inflammatory processes. Once regenerative function and regulation fail, recurrent UTIs become possible.

E-cadherin, UPK-3 and ZO-1 are key factors associated with bladder barrier function and permeability. E-cadherin is an essential factor involved in the cell-cell adhesion necessary to maintain epidermal barrier function [23,24], the uroplakins play important roles in maintaining the urothelial apical surface and are involved in urothelial permeability, and ZO-1 is a tight junction protein expressed in the bladder urothelium [25,26]. Altered bladder barrier function and urothelial morphology were described in animal models of SCI [27]. In this study, decreased E-cadherin, UPK-3, and ZO-1 expression levels indicated the presence of a damaged bladder urothelium and poor barrier function. A damaged bladder wall cannot maintain normal protective functions, and the migration of urinary solutes through the bladder wall may result in further suburothelial inflammation and cell degeneration. Combined with neurogenic inflammation, suburothelial inflammation can further impair basal cell proliferation and apical urothelial function, resulting in a vicious cycle of increased inflammation and poor barrier function [28].

TP63, Ki-67, and BAX are involved in cell differentiation, cell proliferation, and apoptosis. Tryptase, TNF-α, and TGF-β are involved in inflammatory processes. In a bladder that experiences repetitive cycles of destruction and inflammation, cell apoptosis and proliferation appear to be normal physiological responses. In this study, we found that bladder samples form patients with SCI were characterized by protein expressions patterns indicative of accelerated cell apoptosis (increased BAX) and cell proliferation (increased Ki-67), which may indicate the attempt to respond to and defend against infection. A previous animal study indicated that SCI induced inflammatory changes with effects on the innate immune system within the host before, during, and after UTI episodes [12]. Another animal study of acute SCI showed that SCI had negative and severe effects on mitochondrial health in the bladder urothelium [29]. Similar results were demonstrated in the present study, as patients with SCI presented with higher level of TP63, Ki-67, BAX, tryptase, TNF-α, and TGF-β, regardless of the occurrence of a recent rUTI.

Inflammation, cell proliferation, and apoptosis are normal physiological response to bladder insults, such as bacterial invasion or traumatic injury. In cases of acute bladder illnesses (such as infections, tumors, or foreign bodies), this process is typically accelerated [30,31]. In cases of acute immune reactions or cell damage caused by disease or noxious agents, cell apoptosis occurs rapidly. TNF proteins and their receptor play important roles in the extrinsic apoptosis pathway [32]. In a previous animal study examining neurogenic bladder, TNF plays a role in bladder mast cell regulation. Mast cells induced the formation of urothelial lesions, with a concomitant decrease in transepithelial resistance dependent on TNF expression [33]. TGF-β was recently recognized as an inducer of the epithelial-to-mesenchymal transition during fibrotic events in several organs. In a model of the partial obstruction of the bladder outlet, fibrosis was found to be induced by a series of complex pathways involving TGF-β [34,35]. Following cellular damage, proptosis of bladder urothelial cells induces the migration of mast cells, triggering the release of tryptase. Tryptase secreted by mast cells, aggravated bladder damage by activating protease-activated receptor 2, which induced increased barrier dysfunction in the bladder urothelium [36,37].

**Figure 4:**
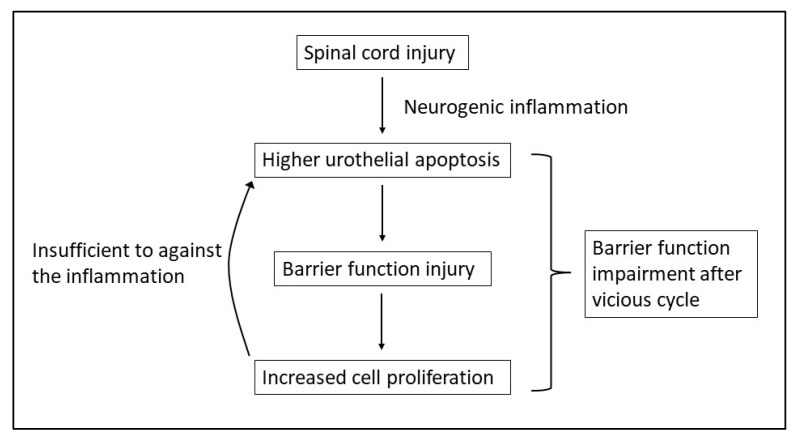
Possible pathway of bladder mucosa injury after chronic SCI.

Our study shows significantly increased expression of inflammatory and apoptotic proteins and proteins associated with urothelial cell proliferation (Ki-67) and differentiation (TP63) in patients with SCI related to controls, but basal cell proliferation (Shh, CD34) and cell differentiation biomarkers (CK-5, CK-14, and CK-20) showed no significant change in SCI bladders. A recent study examining urothelial protein expression in non-SCI patients with persistent rUTI reported the reduced expression levels of Shh, CD34, and TP63. Deficient regenerative abilities are thought to be an underlying cause of recurrent bladder infections, which might result in persistent rUTI in the general population, especially in women [38]. In acute SCI rat models, the umbrella cells of the urothelium were replaced with small, superficial cells from the underlying intermediate cell layer, including cells that were positive for CK-14, CK-5, and TP63 expression. However, 28 days after SCI, the urothelium became morphologically patent, and the proliferative cell number decreased to baseline levels, with patches of small, superficial cells that expressing uroplakins, CK-5, CK-14, and TP63, but lacked CK-20, indicating regions of incompletely differentiated urothelium in chronic SCI bladders [39]. Some materials seem able to reduce the expression of inflammatory cytokines and activate the protection and productive pathway in SCI animal models [40]. The results of this study indicated that the urothelium of patients with chronic SCI retains its regenerative ability but does not respond sufficient to form a well-structured and mature apical cell layer with intact barrier function, possibly due to the presence of persistent neurogenic inflammation. Figure 4 shows the possible pathway of bladder mucosa changes after chronic SCI.

In this study, we did not identify any differences in the bladder barrier function, urothelial differentiation, cell proliferation, apoptosis, or inflammatory protein expression levels between patients with SCI with and without recent rUTI. All patients with SCI included in this study had histories of rUTI, and the only difference between SCI groups was whether they experienced a recent acute UTI episode. The patients’ bladder conditions primarily reflected the presence of neurogenic inflammation and chronic rUTI under condition of chronic SCI rather than an acute UTI episode. These results also indicate that chronic SCI does not result in defects in urothelial regenerative function but instead impair inflammatory, apoptotic, and barrier functions, which may be due to the presence of neurogenic inflammation in chronic SCI. A previous clinical study of detrusor botulinum toxin A injections was able to reduce UTI episodes in patients with chronic SCI, which improved neurogenic inflammation [41]. Our study provides new directions for subsequent treatment of such SCI patients in addition to antibiotics and improving their urination. In the future, more attention should be focused on the elimination of bladder inflammation and improvements in barrier function in patients with chronic SCI to avoid rUTI.

The present study has some limitations. First, a broad range of patient conditions were examined, including various ages, SCI levels, bladder management protocols, specimens obtained, and intervals between SCI duration and bladder biopsy. Our results only represent the bladder conditions at the time of the operation but did not assess sequential changes and lack any evidence of causality. Second, the patients in the control group had different underlying diseases that possibly influenced urothelial protein expression, and protein expression was not standardized according to the different diseases present in this group. Third, the small case number included in the study might potentiate bias in protein expression levels.

## 5. Conclusions

The decreased expression of bladder barrier and junctional proteins and the increased expression of cell apoptosis, proliferation, and differentiation proteins were identified in the bladders of patients with SCI, regardless of whether they experienced recent episodes of rUTI. We did not identify any differences in bladder protein expression levels between the SCI subgroups with and without recent rUTI episodes. These results indicate that chronic SCI bladders present with regenerative functional defects, in association with chronic inflammatory, increased apoptosis, and impairments in differentiation and barrier function. Future treatment for rUTI in chronic SCI patients might focus on the eradication of neurogenic inflammation and improve urothelial barrier function.

## Figures and Tables

**Table 1:** Detailed information regarding antibodies used for western blot.

Antibodies	Company	Catalog Number	Dilution	KDa
E-cadherin	BD Biosciences	BD610181	1:5000	120
ZO-1	Genetex	GTX108627	1:500	190
UPK3	Abcam	ab157801	1:500	32
CK20	Abcam	ab76126	1:2500	48
CK14	Abcam	ab9220	1:1000	58
CK5	Abcam	ab52635	1:1000	62
TP63	Abcam	ab53039	1:1000	77
CD34	Abcam	ab81289	1:10,000	120
SHH	Abcam	ab53281	1:1000	51
Ki-67	Abcam	ab16667	1:1000	358
BAX	Cell Signaling Technology	CST2772S	1:500	20.5
BCL-2	Cell Signaling Technology	CST15071	1:1000	26
P53	Abcam	ab131442	1:1000	53
Tryptase	Merck	mab1222	1:1000	31
TNF-α	Cell Signaling Technology	CST3707S	1:500	17/25
TGF-β	Cell Signaling Technology	CST56E4	1:500	55
GAPDH	Genetex	GTX100118	1:10,000	37

BAX, BCL-2-associated X protein; BCL-2, B-cell lymphoma 2 family protein; CK, cytokeratin; GAPDH, glyceraldehyde-3-phosphate dehydrogenase; Shh, Sonic hedgehog; TGF-β, transforming growth factor-β; TNF-α, tumor necrosis factor-α; TP63, tumor protein p63; UPK3, uroplakin III; UTI, urinary tract infection; ZO-1, zonula occludens-1.

**Table 2:** Baseline urodynamic parameters for patients with spinal cord injury (SCI) and controls.

VUDS	SCI		Total (N = 23)	Control (N = 6)	*p*-Value ^#^	*p*-Value *	*p*-Value **
	rUTI (N = 8)	Non-rUTI (N = 15)					
Pdet	44.63 ± 35.60	27.93 ± 10.18	33.74 ± 23.14	15.00 ± 2.83	0.232	0.131	0.273
Qmax	4.75 ± 5.39	5.40 ± 6.19	5.17 ± 5.8	19.67 ± 14.84	0.805	0.245	0.231
Volume	98.25 ± 127.25	69.93 ± 95.39	79.78 ± 105.51	317.00 ± 124.47	0.552	0.006	0.001
PVR	197.50 ± 165.34	185.33 ± 118.98	189.57 ± 133.19	35.00 ± 60.62	0.840	0.178	0.062
FSF	123.88 ± 91.83	127.73 ± 87.12	126.39 ± 86.7	144.33 ± 75.08	0.922	0.941	0.736
FS	170.00 ± 147.44	173.27 ± 106.21	172.13 ± 118.73	201.67 ± 87.15	0.952	0.920	0.683
Compliance	29.71 ± 31.44	34.65 ± 39.47	32.93 ± 36.21	89.40 ± 50.74	0.763	0.074	0.022
BCI	68.38 ± 52.85	54.93 ± 36.23	59.61 ± 42.03	145.00 ± 73.54	0.478	0.043	0.015
CBC	295.75 ± 232.76	255.27 ± 123.00	269.35 ± 165.09	352.00 ± 71.39	0.587	0.607	0.407
VE	0.31 ± 0.30	0.26 ± 0.26	0.28 ± 0.27	0.88 ± 0.21	0.681	0.005	0.001

*p*-value ^#^: SCI + rUTI vs. SCI + non-rUTI. *p*-value *: SCI + rUTI vs. control. *p*-value **: SCI + non-rUTI vs. control. BCI, bladder contractility index; CBC, cystometric bladder capacity; FS, full sensation; FSF, first sensation of filling; Pdet, maximum detrusor pressure; PVR, post-voiding residual urine volume; Qmax, maximum flow rate; rUTI, recurrent urinary tract infection; SCI, spinal cord injury; VE, voiding efficiency; VUDS, video-urodynamic studies.

**Table 3:** Analysis of bladder samples from patients with SCI compared with that of control group.

	SCI Total (N = 23)	SCI, rUTI (N = 8)	SCI, Non-rUTI (N = 15)	Control (N = 6)	*p*-Value *	*p*-Value ^#^
E-cadherin	0.45 ± 0.40	0.33 ± 0.39	0.32 ± 0.28	0.96 ± 0.31	0.000	0.001
ZO-1	0.42 ± 0.39	0.36 ± 0.40	0.32 ± 0.36	0.73 ± 0.38	0.027	0.089
UPK3	0.42 ± 0.39	0.28 ± 0.22	0.27 ± 0.23	0.97 ± 0.41	0.000	0.000
CK20	0.34 ± 0.22	0.38 ± 0.30	0.34 ± 0.19	0.28 ± 0.21	0.448	0.697
CK14	0.67 ± 0.32	0.77 ± 0.37	0.60 ± 0.34	0.74 ± 0.13	0.408	0.420
CK5	0.27 ± 0.25	0.36 ± 0.35	0.22 ± 0.14	0.26 ± 0.35	0.934	0.614
TP63	0.79 ± 0.50	0.86 ± 0.47	0.89 ± 0.56	0.43 ± 0.19	0.049	0.148
CD34	0.48 ± 0.35	0.40 ± 0.36	0.54 ± 0.39	0.44 ± 0.24	0.791	0.663
Shh	0.57 ± 0.22	0.58 ± 0.27	0.54 ± 0.21	0.63 ± 0.23	0.485	0.721
Ki-67	0.10 ± 0.10	0.11 ± 0.11	0.12 ± 0.11	0.04 ± 0.04	0.011	0.254
BAX	0.34 ± 0.27	0.35 ± 0.25	0.45 ± 0.27	0.06 ± 0.03	0.000	0.004
BCL-2	0.56 ± 0.46	0.48 ± 0.39	0.64 ± 0.56	0.45 ± 0.21	0.537	0.628
P53	0.41 ± 0.27	0.43 ± 0.23	0.48 ± 0.30	0.23 ± 0.18	0.062	0.166
Tryptase	0.78 ± 0.69	1.15 ± 0.80	0.72 ± 0.69	0.44 ± 0.24	0.026	0.115
TNF-α	0.34 ± 0.22	0.40 ± 0.19	0.39 ± 0.19	0.14 ± 0.25	0.009	0.034
TGF-β	0.85 ± 0.44	0.97 ± 0.39	0.94 ± 0.32	0.43 ± 0.54	0.006	0.026

* *p*-value: SCI Total vs. control. ^#^ *p*-value: SCI + rUTI vs. SCI + non-rUTI vs. control. BAX, BCL-2-associated X protein; BCL-2, B-cell lymphoma 2 family protein; CK, cytokeratin; SCI, spinal cord injury; Shh, Sonic hedgehog; TGF-β, transforming growth factor-β; TNF-α, tumor necrosis factor-α; TP63, tumor protein p63; UPK3, uroplakin III; UTI, urinary tract infection; ZO-1, zonula occludens-1.

## Data Availability

Data available on request due to restrictions.

## References

[1] Sekhon L.H., Fehlings M.G. (2001). Epidemiology, demographics, and pathophysiology of acute spinal cord injury. Spine.

[2] Consortium for Spinal Cord Medicine (2006). Bladder management for adults with spinal cord injury: A clinical practice guideline for health-care providers. J. Spinal Cord Med..

[3] Eckert M.J., Martin M.J. (2017). Trauma: Spinal Cord Injury. Surg. Clin. N. Am..

[4] Ku J.H. (2006). The management of neurogenic bladder and quality of life in spinal cord injury. BJU Int..

[5] Manack A., Motsko S.P., Haag-Molkenteller C., Dmochowski R.R., Goehring Jr E.L., Nguyen-Khoa B.A., Jones J.K. (2011). Epidemiology and healthcare utilization of neurogenic bladder patients in a US claims database. Neurourol. Urodyn..

[6] Vizzard M.A. (2006). Neurochemical plasticity and the role of neurotrophic factors in bladder reflex pathways after spinal cord injury. Prog. Brain Res..

[7] Cameron A.P., Rodriguez G.M., Schomer K.G. (2012). Systematic review of urological followup after spinal cord injury. J. Urol..

[8] Gormley E.A. (2010). Urologic complications of the neurogenic bladder. Urol. Clin. N. Am..

[9] Togan T., Azap O.K., Durukan E., Arslan H. (2014). The prevalence, etiologic agents and risk factors for urinary tract infection among spinal cord injury patients. Jundishapur J. Microbiol..

[10] De Ruz A.E., Leoni E.G., Cabrera R.H. (2000). Epidemiology and risk factors for urinary tract infection in patients with spinal cord injury. J. Urol..

[11] Bonkat G., Bartoletti R., Bruyère F., Cai T., Geerlings S.E., Köves B., Schubert S., Wagenlehner F. Urological Infections. EAU Guidelines. Proceedings of the EAU Annual Congress.

[12] Chaudhry R., Madden-Fuentes R.J., Ortiz T.K., Balsara Z., Tang Y., Nseyo U., Wiener J.S., Ross S.S., Seed P.C. (2014). Inflammatory response to Escherichia coli urinary tract infection in the neurogenic bladder of the spinal cord injured host. J. Urol..

[13] Birder L.A. (2006). Role of the urothelium in urinary bladder dysfunction following spinal cord injury. Prog. Brain Res..

[14] Birder L.A., Groat W.C. (2007). Mechanisms of disease: Involvement of the urothelium in bladder dysfunction. Nat. Clin. Pract. Urol..

[15] Birder L.A., Andersson K.E. (2013). Urothelial signaling. Physiol. Rev..

[16] Birder L.A. (2004). Role of the urothelium in bladder function. Scand. J. Urol. Nephrol. Suppl..

[17] Shie J.H., Kuo H.C. (2011). Higher levels of cell apoptosis and abnormal E-cadherin expression in the urothelium are associated with inflammation in patients with interstitial cystitis/painful bladder syndrome. BJU Int..

[18] Chuang F.C., Kuo H.C. (2013). Increased urothelial cell apoptosis and chronic inflammation are associated with recurrent urinary tract infection in women. PLoS ONE.

[19] Krebs J., Wöllner J., Pannek J. (2016). Risk factors for symptomatic urinary tract infections in individuals with chronic neurogenic lower urinary tract dysfunction. Spinal Cord.

[20] Linsenmeyer T.A. (2018). Catheter-associated urinary tract infections in persons with neurogenic bladders. J. Spinal Cord Med..

[21] Mysorekar I.U., Hultgren S.J. (2006). Mechanisms of uropathogenic Escherichia coli persistence and eradication from the urinary tract. Proc. Natl. Acad. Sci. USA.

[22] Shin K., Lee J., Guo N., Kim J., Lim A., Qu L., Mysorekar I.U., Beachy P.A. (2011). Hedgehog/Wnt feedback supports regenerative proliferation of epithelial stem cells in bladder. Nature.

[23] Acharya P., Beckel J., Ruiz W.G., Wang E., Rojas R., Birder L.A., Apodaca G. (2004). Distribution of the tight junction proteins ZO-1, occludin, and claudin-4, -8, and -12 in bladder epithelium. Am. J. Physiol. Ren. Physiol..

[24] Hu P., Meyers S., Liang F.X., Deng F.M., Kachar B., Zeidel M.L., Sun T.T. (2002). Role of membrane proteins in permeability barrier function: Uroplakin ablation elevates urothelial permeability. Am. J. Physiol. Ren. Physiol..

[25] Wu X.R., Kong X.P., Pellicer A., Kreibich G., Sun T.T. (2009). Uroplakins in urothelial biology, function, and disease. Kidney Int..

[26] Zwaans B.M.M., Carabulea A.L., Bartolone S.N., Ward E.P., Chancellor M.B., Lamb L.E. (2021). Voiding defects in acute radiation cystitis driven by urothelial barrier defect through loss of E-cadherin, ZO-1 and Uroplakin III. Sci. Rep..

[27] Apodaca G., Kiss S., Ruiz W., Meyers S., Zeidel M., Birder L.A. (2003). Disruption of bladder epithelium barrier function after spinal cord injury. Am. J. Physiol. Ren. Physiol..

[28] Homma Y., Ueda T., Tomoe H., Lin A.T.L., Kuo H.C., Lee M.H., Lee J.G., Kim D.Y., Lee K.S. (2009). Interstitial cystitis guideline committee. Clinical guidelines for interstitial cystitis and hypersensitive bladder syndrome. Int. J. Urol..

[29] Kullmann A.F., Truschel S.T., Wolf-Johnston A.S., McDonnell B.M., Lynn A.M., Kanai A.J., Kessler T.M., Apodaca G., Birder L.A. (2019). Acute spinal cord injury is associated with mitochondrial dysfunction in mouse urothelium. Neurourol. Urodyn..

[30] Norbury C.J., Hickson I.D. (2001). Cellular responses to DNA damage. Annu. Rev. Pharmacol. Toxicol..

[31] Jiang Y.H., Liu H.T., Kuo H.C. (2015). Urothelial dysfunction and chronic inflammation in patients with spinal cord injuries at different levels and correlation with urodynamic findings. Neurourol. Urodyn..

[32] Chen M.C., Blunt L.W., Pins M.R., Klumpp D.J. (2006). Tumor necrosis factor promotes differential trafficking of bladder mast cells in neurogenic cystitis. J. Urol..

[33] Chen M.C., Mudge C.S., Klumpp D.J. (2006). Urothelial lesion formation is mediated by TNFR1 during neurogenic cystitis. Am. J. Physiol. Ren. Physiol..

[34] Wang W., Wang X., Chun J., Vilaysane A., Clark S., French G., Bracey N.A., Trpkov K., Bonni S., Duff H.J. (2013). Inflammasome-independent NLRP3 augments TGF-β signaling in kidney epithelium. J. Immunol..

[35] Hughes J.F.M., Sexton S.J., Jin H., Govada V., Purves J.T. (2017). Bladder fibrosis during outlet obstruction is triggered through the NLRP3 inflammasome and the production of IL-1β. Am. J. Physiol. Ren. Physiol..

[36] Wu Z., Li Y., Liu Q., Liu Y., Chen L., Zhao H., Guo H., Zhu K., Zhou N., Chai T.C. (2019). Pyroptosis engagement and bladder urothelial cell-derived exosomes recruit mast cells and induce barrier dysfunction of bladder urothelium after uropathogenic E. coli infection. Am. J. Physiol. Cell Physiol..

[37] Akiyama Y., Maeda D., Morikawa T., Niimi A., Nomiya A., Yamada Y., Igawa Y., Goto A., Fukayama M., Homma Y. (2018). Digital quantitative analysis of mast cell infiltration in interstitial cystitis. Neurourol. Urodyn..

[38] Jhang J.F., Lin T.Y., Ho H.C., Jiang Y.H., Hsu Y.H., Birder L.A., Kuo H.C. (2021). Deficits of urothelial cell proliferation, cytoskeleton, and barrier function protein expressions in patients with recurrent and persistent urinary tract infections. Low. Urin. Tract Symptoms..

[39] Kullmann F.A., Clayton D.R., Ruiz W.G., Wolf-Johnston A., Gauthier C., Kanai A., Birder L.A., Apodaca G. (2017). Urothelial proliferation and regeneration after spinal cord injury. Am. J. Physiol. Ren. Physiol..

[40] Wojdasiewicz P., Poniatowski Ł.A., Turczyn P., Frasuńska J., Paradowska-Gorycka A., Tarnacka B. (2020). Significance of Omega-3 Fatty Acids in the Prophylaxis and Treatment after Spinal Cord Injury in Rodent Models. Mediators Inflamm..

[41] Jia C., Liao L.M., Chen G., Sui Y. (2013). Detrusor botulinum toxin A injection significantly decreased urinary tract infection in patients with traumatic spinal cord injury. Spinal Cord.

